# Analysis of the demographic profile and influences on the prognosis of COVID-19 treated at a public hospital

**DOI:** 10.3205/dgkh000526

**Published:** 2024-12-16

**Authors:** Júlia França da Silva, Amanda Moura Ferreira, Bianca Bastos Redressa da Silva, Clara Thuany Pellis Mizusaki, Isabela Catini Bautz, Emilene Cristine Izu Nakamura Pietro, Lucas Marques da Costa Alves, Rodrigo Cardoso Oliveira, Roosevelt da Silva Bastos, Paulo Sérgio da Silva Santos

**Affiliations:** 1 Department of Surgery, Stomatology, Pathology, and Radiology, Bauru School of Dentistry, University of São Paulo, Bauru, São Paulo, Brazil; 2 Department of Biological Sciences, Bauru School of Dentistry, University of São Paulo, Bauru, São Paulo, Brazil; 3 State Hospital of Bauru, Foundation for Medical and Hospital Development, Bauru, São Paulo, Brazil; 4 Department of Pediatric Dentistry, Orthodontics and Public Health, Bauru School of Dentistry, University of São Paulo, Bauru, São Paulo, Brazil

**Keywords:** COVID-19, epidemiology, comorbidity, public hospital, prognosis

## Abstract

**Objectives:**

The objectives of this retrospective cross-sectional study were to evaluate the demographic profile and aggravating factors in the prognosis of patients with COVID-19 in the years 2020 and 2022.

**Methods:**

From the analysis of medical records, data were collected on age, sex, race, and municipality of residence, as well as dates of onset of symptoms, positive test result and length of hospitalization. Demographic data were analyzed qualitatively, while information on the presence of comorbidities and their influence on length of hospitalization, disease outcome and need for ICU admission were assessed using Pearson's correlation test. Student's t-test was used to compare the two analyzed moments, relating the age of patients and the progression of the disease from the onset of symptoms to the positive test result and finally clinical outcome.

**Results:**

Among the most common comorbidities, hypertension had an influence on prognosis, as did the age and sex of the patients, with a higher prevalence of male patients over the age of 64.

Vaccinated patients had a better prognosis when compared to those who were unvaccinated.

**Conclusion:**

The findings highlight the continued need for public health strategies, including vaccination against COVID-19, risk monitoring and measures for vulnerable groups.

## Zusammenfassung

**Ziele:**

Mit einer retrospektiven Querschnittsstudie sollten das demografische Profil und erschwerende Faktoren für die Prognose von COVID-19-Patienten in den Jahren 2020 und 2022 analysiert werden.

**Methode:**

Anhand der Analyse von Krankenakten wurden Daten zu Alter, Geschlecht, ethnischer Zugehörigkeit und Wohnort sowie zu Symptombeginn, positivem Testergebnis und Dauer des Krankenhausaufenthalts erhoben. Demografische Daten wurden qualitativ analysiert, während Informationen über das Vorhandensein von Begleiterkrankungen und deren Einfluss auf die Dauer des Krankenhausaufenthalts, den Krankheitsverlauf und die Notwendigkeit einer Aufnahme auf die Intensivstation mit dem Pearson-Korrelationskoeffizienten bewertet wurden. Der t-Test nach Student wurde verwendet, um die beiden untersuchten Zeitpunkte zu vergleichen und das Alter der Patienten sowie den Krankheitsverlauf vom Symptombeginn über das positive Testergebnis bis hin zum klinischen Ausgang zu analysieren.

**Ergebnisse:**

Bluthochdruck gehörte zu den häufigsten Begleiterkrankungen und hatte Einfluss auf die Prognose, ebenso wie Alter und Geschlecht der Patienten mit einer höheren Prävalenz bei männlichen Patienten über 64 Jahren. Geimpfte Patienten wiesen eine bessere Prognose als ungeimpfte Patienten auf.

**Schlussfolgerung:**

Die Ergebnisse unterstreichen die permanente Notwendigkeit von Strategien im Bereich ÖPublis Health einschließlich Impfungen, Risikobewertungen und Maßnahmen für gefährdete Gruppen im Kampf gegen COVID-19.

## Introduction

Infections with SARS-CoV-2 can be asymptomatic or cause respiratory infections ranging from mild to severe, and may even lead to death [1]. On March 11, 2020, the World Health Organization (WHO) declared COVID-19 a pandemic, due to the increasing number of infections and the mortality rate worldwide [2]. The first confirmed cases of COVID-19 in Brazil occurred in February 2020, and since then, the number of infections in the country has continued to grow exponentially, with over thirty million confirmed cases, according to the Coronavirus Panel [3].

The clinical consequences of infection vary among individuals, and with the rise in hospitalizations and deaths, various factors have been scrutinized to identify risk and protective factors for COVID-19 [4], [5], [6]. Much discourse has centered on attributes that might predispose a patient to a worse prognosis upon detection of the COVID-19 virus, including prioritized risk groups during vaccination campaigns. Among the factors under consideration are age, gender, occupation, and the presence of comorbidities, ranging from underlying conditions to patients requiring specialized treatments such as hemodialysis [7], [8]. Vaccination, initiated in Brazil on January 17, 2021, emerged as the pivotal strategy in curtailing mortality and morbidity during the pandemic [9]. Regarding the mechanism of action of vaccines administered to the populace, recent studies indicate that the elicitation of immunological memory by the vaccine can significantly impact not only virus infection but also avert reinfection [10]. Even after the control of COVID-19 improved, new cases and mutations remain possible, making it extremely important to analyze conditions that may lead to a worse prognosis and factors that can assist the healthcare system in combating the virus. Therefore, this article aims to collect and analyze data to examine the profile of patients hospitalized in DRS VI of Bauru, as well as the factors that contributed to patients having a better or worse prognosis.

## Materials and methods

### Bioethical considerations

The project was developed in accordance with the Declaration of Helsinki (1964) and the Resolution No. 466 of the National Health Council (Ministry of Health of Brazil, 2012). The study protocol was approved by Institutional Research Ethics Committee (67338423.8.0000.5417).

### Study characterization

This study retrospective cross-sectional study was conducted with the objective of comprehensively understanding the potential impacts of comorbidities and vaccination coverage on the population during the COVID-19 pandemic. It evaluated demographic and health profiles of patients who received treatment for COVID-19 infection at Hospital Estadual de Bauru, a public hospital in Brazil, during two distinct phases of the pandemic.

From the analysis of hospitalized patients’ records (E-pront^®^), data such as gender, age group, sex, ethnicity, and city of origin were examined to conduct the demographic analysis. Subsequently, data pertaining to the progression of the disease were scrutinized to analyze the viral cycle and the efficiency of the mandatory notification system implemented by the unified healthcare system, encompassing the dates of symptom onset, positive testing, and hospital admission.

Subsequently, data regarding the presence of comorbidities and their potential influences on disease progression were collected through the analysis of the length of hospital stay, whether ICU admission was required or not and its respective duration, as well as the outcome of hospitalization, assessing whether the patient died or was discharged from the hospital.

### Sample characterization

The sample consisted of 764 patients in the year 2020 and 27 patients in the year 2022, totaling 791 patients, with or without comorbidities, who were admitted to the State Hospital of Bauru in the first six months of the pandemic and the last six months, corresponding to March 2020–September 2020 and May 2022–November 2022, respectively. As exclusion criteria, only patients diagnosed with other types of respiratory diseases other than COVID-19 and those under 18 years of age were not included in the sample.

### Statistical analysis

A database covering all information obtained was organized in a Microsoft Office Excel 2019 spreadsheet to tabulate demographic data. When analyzing the influence of age and time between onset of symptoms, positive test and hospitalization on the evolution of the disease, Pearson’s correlation test (P<0.05) and the Chi-squared test (P<0.05) were used. To compare data between the years 2020 and 2022, the relationship between age of the patients and the length of hospital stay was examined using Student's t-test (P<0.05). The influence of comorbidities on the occurrence of deaths was also analyzed using the Student's t-test (P<0.05).

## Results

### Demographic analysis

764 medical records for the year 2020 were analyzed. 398 (52.09%) patients were male, 540 (70.68%) were white, 417 (54.58%) were from the city of Bauru, and the average age of hospitalized patients was 64.3 years. In the year 2022, 27 medical records were evaluated; 17 (62.96%) patients were male, 18 (66.66%) were white, 16 (59%) were from the city of Bauru, and the average age of hospitalized patients was 64 years. Regarding the vaccine dose, only two patients had been vaccinated, the first having been vaccinated with 3 doses and the second with 4 doses.

In 2020, 113 of 209 patients (54.06%) who required intensive care and were admitted to the ICU were male. Likewise, 202 of 364 (55.50%) deaths during this period were male. On the other hand, in 2022, 3 of 5 patients (60%) admitted to the ICU were female, a situation that was repeated with regard to deaths, with 5 of the 9 (55%) patients being female. However, in the periods analyzed, the highest prevalence of hospitalized patients was male, with 398 (52.09%) patients in 2020 and 17 (62.96%) in 2022.

### Lenght of stay and disease progression

In 2020, the average length of stay was 10.9 days, while in 2022 it was 17.8 days.

In relation to the length of stay in the ICU, both in 2020 and in 2022, it was observed that the longer the stay in the ICU, the greater the total number of days of hospitalization.

The analyses of the years 2020 and 2022 showed that the longer the time between the onset of symptoms and hospitalization, as well as between the date of positive result and hospitalization, the shorter the length of hospitalization.

### Influence of comorbidities on prognosis

The length of hospital stay did not vary significantly when the presence or absence of comorbidities was tested in the two periods evaluated. Thus, there was an average hospitalization period of 10.9 days for patients with some type of comorbidity in both evaluated periods.

In view of this, it was observed that the presence of at least one comorbidity contributed to a worse prognosis of the disease, so that out of the 374 patients who required ICU admission, 316 (84.5%) had at least one type of comorbidity. Similarly, out of 214 patients who died, 185 (86.4%) had at least one type of comorbidity.

It was also observed that of the comorbidities observed, hypertension was the most frequent in both evaluated periods, with 416 (52.59%) of 791 patients hospitalized in the two periods having this comorbidity.

Overall, the number of patients who required ICU admission was 374, with 228 (60.96%) patients having arterial hypertension, associated or not with any other comorbidity. Analyzing the number of patients who died, 138 out of 214 (64.48%) patients had arterial hypertension, associated or not with any other comorbidity. The other comorbidities were not relevant to the severity of the cases.

## Discussion

The study presented an analysis of data obtained from medical records of patients who were hospitalized in a public hospital during the initial and final months of the COVID-19 pandemic. As seen in other studies, the data showed that male patients had a worse prognosis. This is evident not only in the difference between the number of female and male patients who required ICU admission but also in relation to deaths, especially in the year 2020. According to Mukherjee and Pahan [11], this difference can be multifactorial. Hypotheses have been raised regarding findings of elevated levels of ACE2 and TMPRSS2 in men [12], [13], differences in hormonal influence on the immune response [14], and even differences in behavior, such as tobacco use, which may lead to the development of comorbidities [8].

The age of patients was one of the factors identified as significant predictor in the prognosis of COVID-19, as seen in this study where patients over 60 years old had higher rates of ICU admission and deaths. Studies aiming to evaluate the characteristics of patients infected with the SARS-CoV-2 [15], [16] virus consistently demonstrated that older individuals tend to face a substantially higher risk of developing severe complications and even mortality compared to younger people [17]. Advanced age is associated with a potentially weakened immune system (immunosessence), which may result in a less effective immune response against the virus. Additionally, pre-existing medical conditions, more common in older individuals, can also contribute to a more challenging clinical outcome. Understanding these relationships is crucial for identifying more effective care strategies, especially for the elderly population, in order to minimize the adverse impacts of COVID-19.

Observational studies suggest that hypertension is also associated with a worse prognosis of COVID-19 [18], [19], [20]. Hypertensive patients infected with SARS-CoV-2 have a higher risk of developing severe forms of the disease [21], including respiratory failure, acute respiratory distress syndrome, and death, as seen in this study, where more than half of the deceased patients had arterial hypertension, whether associated with other comorbidities or not. When evaluating the data related to the length of hospital stay for both groups of patients, some findings deserve discussion. It was observed that, regardless of the stage of the pandemic, the longer the time between the onset of symptoms and hospitalization, as well as between the date of the positive result and hospitalization, the shorter the length of stay. In other words, the longer the diagnosis took, the shorter the hospital stay, regardless of the outcome. This situation suggests the importance of promptness in diagnosis and clinical intervention to ensure effective management of the disease and potential complications.

Comparing the population of 2020 and 2022 in this study as well as in other observational studies [22], [23], we noticed a significantly lower number in the cases, which can be attributed to the beginning of COVID-19 vaccination coverage. Despite this smaller number of hospitalized individuals, the clinical characteristic regarding diagnosis time/severity/and hospitalization remained, with the same profile as in the first period of the pandemic. The study consists of a curated analysis of the medical records of patients admitted to a public hospital in the interior of São Paulo at the beginning and end of the COVID-19 pandemic, showing important correlations between the clinical stage, demographic factors, and pre-existing medical conditions. Being male is associated with more severe stages, based on biological and behavioral factors, such as the expression of certain receptors and hormonal differences, supporting the idea of carrying out specific approaches for each group of patients. Furthermore, advanced age and the presence of comorbidities, especially high blood pressure, were found to be predictors for complications and deaths, making greater vigilance and care for this group of patients extremely important. 

It was seen that vaccines against COVID-19 were effective, reflecting results from other research, in view of the large reduction observed in hospitalizations in the last six months of the pandemic, a period when vaccination coverage among the population was already well advanced.This attests to the crucial and protective role of vaccination in preventing diseases and its complications, thus offering hope to control possible advances of the disease in the future, with new mutations of the virus. 

The results of the study point to a continued need for public health strategies, including the promotion of widespread vaccination, close monitoring of risk factors, and the implementation of specific measures for more vulnerable groups. These measures can facilitate risk mitigation and reduce adverse effects of COVID-19, in addition to furthering the fight against this global disease.

## Conclusions

The study highlights important correlations between demographic factors, preexisting medical conditions, and clinical outcomes.

Regarding the demographic profile, there was a higher prevalence of patients from the city where the hospital is located, and male patients in the 65-year-old age group. Much of this can be attributed to the fact that patients in this age group tend to have at least one type of comorbidity, along with the presence of harmful habits.

The presence of hypertension, whether associated with other comorbidities or not, was observed to worsen the course of COVID-19 infection. It was present in more than half of the patients who required ICU admission and more than half of the patients in this study who died.

The severity of the disease assessed by the length of hospital stay and the interval between diagnosis and hospitalization did not show significant differences in the two periods evaluated, but brought up the issue that the longer it took to obtain the diagnosis, the shorter the hospital stay would be. This situation could be be explained by the attenuation of symptoms as the host's contamination period passed.

## Notes

### Competing interest

The authors declare that they have no competing interests.

### Ethical approval

The study was approved by the Human Research Ethics Committee of Bauru Schoolof Dentistry of the University of Sao Paulo, Brazil (CAAE 67338423.8.0000.5417).

### Funding sources

The study was supported by a project grant from Ministry of Health (PET-SAÚDE 2022/2023), Secretariat of Management Work and Education in Health (SMWEH).

### Authors’ ORCID

- da Silva JF: 0000-0003-1314-8795
- Ferreira AM: 0000-0001-5371-5753
- Bastos Redressa da Silva B: 0009-0003-0615-422X
- Thuany Pellis Mizusaki C: 0009-0009-8643-9797
- Catini Bautz I: 0000-0003-3843-8764
- Izu Nakamura Pietro EC: 0000-0002-4113-3980
- Marques da Costa Alves L: 0000-0001-9018-6395
- Cardoso Oliveira R: 0000-0001-5785-2261
- da Silva Bastos R: 0000-0001-5051-1210
- da Silva Santos PS: 0009-0005-0687-8839
